# Socioeconomic and Medical Vulnerabilities Among Syrian Refugees with Non-communicable Diseases Attending Médecins Sans Frontières Services in Irbid, Jordan

**DOI:** 10.1007/s10903-022-01408-7

**Published:** 2022-10-22

**Authors:** Antonio Isidro Carrion-Martin, Ahmad Alrawashdeh, Georgios Karapanagos, Refqi Mahmoud, Nashaat Ta’anii, Mais Hawari, Stefanie Dittmann, Luna Hammad, Geertje Huisman, Mark Sherlock, Amulya Reddy

**Affiliations:** 1 Médecins Sans Frontières, Amsterdam, The Netherlands; 2 Médecins Sans Frontières, Amman, Jordan; 3 Jordan Ministry of Health, Amman, Jordan; 4 Faculty of Applied Medical Sciences, Jordan University of Science and Technology, Irbid, Jordan

**Keywords:** Non-communicable disease, Refugees, Medical vulnerabilities, MSF, Healthcare access

## Abstract

Non-communicable diseases (NCDs) are high-prevalence health problems among Syrian refugees. In 2014, Médecins Sans Frontières (MSF) identified unmet NCD care needs and began providing free-of-charge services for Syrian refugees in Irbid, Jordan. This study aimed to describe current socioeconomic and medical vulnerabilities among MSF Irbid Syrian refugee patients and their households and raise awareness of their ongoing health needs that must be addressed. A cross-sectional survey among Syrian refugees attending MSF NCD services in Irbid Governorate, Jordan was conducted by telephone interviews in January 2021 to query sociodemographic characteristics, economic situation, self-reported NCD prevalence, and Ministry of Health (MoH) policy awareness. Descriptive analysis of indicators included proportions or means presented with 95% confidence intervals. The survey included 350 patient-participants in 350 households and 2157 household members. Mean age was 28.3 years. Only 13.5% of household members had paid or self-employed work; 44% of households had no working members. Mean monthly income was 258.3 JOD (95%CI: 243.5–273.1) per household. Mean expenditures were 320.0 JOD (95%CI: 305.1–334.9). Debt was reported by 93% of households. NCD prevalence among adults was 42% (95%CI: 40–45). Hypertension was most prevalent (31.1%, 95%CI: 28.7–33.7), followed by diabetes (21.8%, 95%CI: 19.7–24.1) and cardiovascular diseases (14.4%, 95%CI: 12.6–16.4). Only 23% of interviewees were aware of subsidized MoH rates for NCD care. Twenty-nine percent stated they will not seek MoH care, mainly due to the unaffordable price. Our findings highlight increased vulnerability among MSF Irbid Syrian refugee NCD patients and their households, including: an older population; a high percentage of unemployment and reliance on cash assistance; higher proportion of households in debt and a high number of households having to resort to extreme coping mechanisms when facing a health emergency; and a higher proportion of people with multiple comorbid NCDs and physical disability. Their awareness of subsidised MoH care was low. MoH care is expected to be unaffordable for many. These people are at increased risk of morbidity and mortality. It is vital that health actors providing care for Syrian refugees take action to reduce their risk, including implementing financial support mechanisms and free healthcare.

## Background

Since its start in 2011, the Syrian civil war has forced over 5.6 million refugees into neighbouring countries [1], with 95% not living in camps but in urban, peri-urban and rural areas. Jordan has received 11.9% of these refugees. Among Syrian refugees in Jordan, 2018 prevalence of chronic diseases was reported as 29% [2] and a 2019 systematic review noted non-communicable diseases to be the most common health problems [3]. A 2019 Jordan National Stepwise Survey showed comparable hypertension prevalence of 22% and diabetes 20% among Jordanians and Syrians [4].

The Jordan Ministry of Health (MoH) provided Syrian refugees with free healthcare until 2014, when it implemented subsidized uninsured Jordanian rates. In late 2014, Médecins Sans Frontières (MSF) began providing free-of-charge non-communicable diseases (NCD) care for Syrian refugees living in Irbid Governorate, which registers 136,498 Syrians living in non-camp circumstances [1]. MSF’s primary care level clinic received Syrian refugees and vulnerable Jordanians with diabetes, hypertension, cardiovascular diseases, and chronic respiratory diseases (asthma, chronic obstructive pulmonary disease (COPD)) in a model of care that also provided ancillary laboratory, health education, physiotherapy and mental health support services.

In 2019, MSF began planning handover of its Jordan programme, aiming to transfer patients by the end of 2021 to MoH and other partners. A population-based survey in Irbid Governorate was planned for 2020 since a 2017 MSF survey had shown 22% NCD prevalence, with 44% multi-morbidity among NCD patients, 23% of whom did not seek care due to unaffordability [5].

Due to the COVID pandemic, however, this changed to a telephone survey in 2021. The survey aimed to describe current socioeconomic and medical vulnerabilities among MSF Irbid Syrian refugee NCD patients and their households and raise awareness of their ongoing healthcare needs that must be addressed.

## Methods

This was a cross-sectional survey among Syrian refugees attending MSF NCD services in Irbid Governorate, Jordan, using telephone interviews and electronic data collection tools. Telephones are in wide use and incoming calls do not incur charges in Jordan. Telephone interviews were conducted between 14 January and 21 January 2021. There were 3581 patients in the cohort, 328 of whom receive home-based care given multiple morbidities and poor mobility.

### Inclusion Criteria and Definitions

A person was included in the study if they satisfied all the following criteria: (i) NCD patient in the MSF cohort; (ii) Syrian refugee arrived in Jordan in 2012 or after and (iii) resident in Irbid for more than six months prior to the interview.

A household is a person or group of people who live in the same housing unit and share living arrangements (e.g., meals, resources). A permanent household member is a person that resides regularly in the household and is mainly dependent on it. NCDs queried included diabetes, hypertension, cardiovascular diseases, chronic respiratory diseases, thyroid disease and/or cancer. The medical severity score (MSS) classifies patients by clinical criteria for the queried NCDs. The MSS ranges between zero and three, with zero indicating stable/controlled medical status and scores between one and three indicating unstable/uncontrolled medical status (MSS = 3 reflects home visit service eligibility). Physical disability indicates need for assistance during routine daily activities.

The distribution of MSF Irbid cohort NCD patients by medical severity score at the time of data collection is shown in Table 1.

**Table 1:** Distribution of NCD patients by Medical Severity Score

Medical severity score	n	%
0	1746	53.92
1	295	9.11
2	919	28.38
3	278	8.59
Total	3238	100.00

### Sampling and Sample Size

We used simple random sampling of MSF Irbid Syrian NCD patients, who were then divided into two groups: (i) stable (MSS = zero) and (ii) unstable (MSS = one to three). Sampling was done by Irbid clinic staff.

Sample size was calculated to estimate the socioeconomic indicators (at the household level) and knowledge of recent MoH policy changes (among MSF Syrian NCD patients). We used a conservative approach, considering 50% anticipated prevalence and 5% precision, and arrived at a sample size of 342 patients-participants and their households. We inflated the random selection for a high non-response rate of 40%, which resulted in 484 patients-participants and their households.

### Data Collection and Analysis

The household interviews followed a 4-part questionnaire: (i) sociodemographic characteristics (all household members)—age, sex, educational level, employment, UNHCR and Ministry of Interior (MOI) registration; (ii) economic situation (household level, in the last month)—income, non-salary income, non-monetary assistance, household expenditure, debt, resort to financial adaptative mechanisms when encountering an urgent health problem; (iii) self-reported NCD prevalence (all household members)—hypertension, diabetes, cardiovascular disease, chronic respiratory disease, thyroid disease and cancer; and (iv) MoH policy awareness (for MSF NCD patients)—knowledge of policy changes and willingness to seek MoH care after MSF closes. The questionnaire included items not discussed in this paper.

The survey questionnaire was designed in English, translated to Arabic and back translated to English for verification. Interviewers were medical students who received a 2-day training on the study, questionnaire and daily call sheet. Interviewers were trained on potential bias, respectful behaviour, communication skills, and how to start a telephone interview, explaining the purpose of their call [6]. The questionnaire was piloted (20 interviews) and some small changes were subsequently made. Data were entered using a mobile data collection system with the open-source toolbox KOBO (https://www.kobotoolbox.org/).

All data collected were anonymized (no names, exact location or telephone numbers were collected); electronic files were stored password-protected by MSF. Data cleaning and analysis were conducted using STATA 16 (StataCorp, College Station, TX, USA). Descriptive analysis of indicators included proportions or means, which are presented with 95% confidence intervals (95%CI). Differences in proportions were measured using Pearson χ^2^ test; differences in means were measured using Student t test for parametric variables and Kruskal-Wallis quality-of-populations rank test for non-parametric variables (p-values are presented).

## Results

Of 484 randomly selected NCD patients, 390 were reached by the clinical team (n = 94, 19% of participants were not reached on the phone). Fifteen (3%) subsequently did not consent. Among the 375 who accepted, 9 were not reached by the survey team and 13 did not meet inclusion criteria. Data were lost for 3 patients (data tool errors). The survey therefore included 350 patient-participants in 350 households and 2157 household members, with characteristics as shown in Table 2.

**Table 2:** Characteristics of Syrian participants and household members.

	MSF patients n=350		Entire sample n= 2157	
	n (%)	95% CI	n (%)	95% CI
Age				
≤17	4 (1.1)	0.4–3.0	842 (39.0)	35.1–42.3
18–39	16 (4.6)	2.8–7.3	673 (31.2)	29.9–33.0
40–59	178 (50.9)	45.6–56.1	388 (18.0)	16.1–19.8
≥60	152 (43.4)	38.3–48.7	254 (12.0)	10.1–13.7
Gender				
Male	152 (43.4)	38.3–48.7	1029 (47.7)	45.2–49.5
Female	198 (56.6)	51.3–61.7	1128 (52.3%)	(50.2–54.4).
Education				
No formal education	76 (21.7)	17.7–26.4	8.3	7.2–9.6
Primary	209 (59.7)	54.5–64.7	1352 (62.7)	61.3–64.1
Secondary/post-secondary	65 (18.6)	14.8–23.0	121 (9.2)	7.8–10.9
Work status				
Not working	316 (90.3)	86.7–93.0	1877 (87.0)	86.7–88.6
Working (paid or self-employed)	34 (9.7)	7.0–13.3	280 (13.0)	11.5–14.7
Legal status				
UNHCR	337 (96.3)	93.7–97.8	2071 (96.0)	95.1–96.8
MOI card	343 (98)	95.9–99.0	2026 (93.9)	92.8–94.9
UNCHR & MOI card	333 (95.1)	92.3–97.0	1993 (92.4)	91.2–93.4
Prevalence of one or more NCDs	350 (100)	–	601 (27.9)	26.0–29.8
NCD prevalence				
Diabetes	210 (60.0)	54.8–65.0	184 (25.9)	22.8–29.2
HTN	278 (79.4)	74.9–83.4	238 (33.5)	30.1–37.0
CVD	127 (36.3)	31.4–41.5	99 (13.9)	11.6–16.7
Respiratory	41 (11.7)	8.7–15.5	104 (4.8)	4.0.–5.8
Thyroid	31 (8.9)	6.3–12.3	63 (2.9)	2.3–3.7
Cancer	5 (1.4)	0.6–3.4	16 (0.7)	0.5–1.2

### Demographic and Economic Characteristics

Mean household member age was 28 years (range: 1–90, standard deviation (SD): 21.3). Females constituted 52% of individuals (1128/2157, 95%CI: 50–54). The proportion of adults (≥18 years) was 61% (1315/2157, 95%CI: 59–63) and those aged >65 years 7.5% (162/2157, 95%CI: 6.5–8.7). Mean household size was 6.2 members (range: 1–20, SD: 3.0). A total of 25 (7.2%) households arrived before 2012 and only 24 (6.9%) households arrived between 2014 and 2018. Households have been in Jordan for a mean 8.4 years (95% CI, 8.3–8.5).

Only 280 of 2157 (13%, 95%CI: 12–14) individuals were in paid work (n = 273) or self-employed (n = 7) (Table 3). Forty-four percent of households (153/350, 95%CI: 39–49) had no working members; 39% (137/350, 95%CI: 34–44) had only one working member.

**Table 3:** Work status of adults (18–65 years)

Work status (adults 18–65 years)	n	%	95% CI
Working for wages or salary	265	23.0	20.6–25.5
Self-employed	7	0.6	0.3–1.3
Available and actively looking for work	156	13.5	11.7–15.6
In school/training	86	7.5	6.1–9.1
Doing home duties	396	34.4	31.7–37.1
Not working due to chronic health condition	227	19.7	17.5–22.1
Retired	7	0.6	0.3–1.3
Don’t know	9	0.8	0.4–1.5

Most individuals were registered with UNHCR (n = 2071, 96%, 95%CI: 95–97) and had an MOI card (n=2026, 94%, 95%CI: 93–95). Full legal status (UNHCR and MOI) was reported for 92% (n = 1993, 95%CI: 91–93). Main reasons for not registering with UNHCR were ‘not needed’, ‘not applicable/other nationality’ and ‘UNHCR is closed’; ‘lack of time’ was the main reason for lack of MOI registration.

Mean monthly income was 258 JOD (range: 0–2004, SD: 169) per household and 48 JOD (range-0–501, SD: 39) for each member, while mean expenditures were 320 JOD (range: 305–335, SD: 8) and 62 JOD (range: 0–300, SD: 2) respectively. Debt was reported by 93% of households (324/350, 95%CI: 89–95). Main sources of household income were UNHCR cash assistance (184/350, 53%, 95%CI: 47–58) and work (149/350, 43%, 95%CI: 37–48).

Seventy percent (243/350, 95%CI: 64–74) of households reported that they resorted to financial adaptive approaches for urgent health problems as detailed in Table 4.

**Table 4:** Adaptive responses to urgent health problems in families of Syrian refugees attending Médecins Sans Frontières NCD services in Irbid Governorate, Jordan, 2021.

Responses	n	%	95%CI
Borrow money from friend/relative	209	60	54–65
Reduce standard household spending	207	59	54–64
Turn down work or missing workdays	107	31	26–36
Sell household assets	63	18	14–23
Borrow money from usurer	12	3.4	2.0–6.0

### Non-communicable Diseases and Physical Disability

Twenty-eight percent of household members (601/2157, 95%CI: 26–30) reported having one or more NCDs, including diabetes, hypertension, cardiovascular diseases, chronic respiratory disease, thyroid disease, and/or cancer. Adult (≥18 years) NCD prevalence was 42% (556/1.315, 95%CI: 40–45). Women had higher prevalence than men (46%, 95%CI: 42–50 and 38%, 95%CI: 34–42, p = 0.003).

Among adults, hypertension was most prevalent (31%, 95%CI: 29–34), followed by diabetes (22%, 95%CI: 20–24) and cardiovascular disease (14%, 95%CI: 13–16). Among minors, chronic respiratory disease was most prevalent NCD (3.1%, 95%CI: 2.1–4.5) (Table 5). The prevalence of most NCDs increased with age; this was most strongly observed for diabetes, hypertension and cardiovascular conditions. The prevalence of most NCDs was higher in women than in men (Table 5).

**Table 5:** NCD prevalence among Syrian refugees attending Médecins Sans Frontières NCD services in Irbid Governorate, Jordan, 2021

	Entire population (n=2157)		Minors (<18 years) (n=842)		Adults (≥18 years) (n=1315)		Women (n=711)		Men (n=604)	
	n (%)	95%CI	n (%)	95%CI	n (%)	95%CI	n (%)	95%CI	n (%)	95%CI
Diabetes	296 (14)	12–15	9 (1.1)	0.6–2.0	287 (22)	20–24	184 (26)	23–29	103 (17)	14–20
Hypertension	410 (19)	17–21	1 (0.1)	0.0–0.8	409 (31.1)	29–34	238 (33.5)	30–37	171 (28)	25–32
Cardiovascular diseases	198 (9.2)	8.0–10	9 (1.1)	0.6–2.0	189 (14)	13–16	99 (13.9)	12–17	90 (15)	12–18
Respiratory diseases	104 (4.8)	4.0–5.8	26 (3.1)	2.1–4.5	78 (5.9)	4.8–7.3	48 (6.8)	5.1–8.8	30 (5.0)	3.5–7.0
Thyroid diseases	63 (2.9)	2.3–3.7	1 (0.1)	0.0–0.8	62 (4.7)	3.7–6.0	52 (7.3)	5.6–9.5	10 (1.7)	0.9–3.1
Cancer	16 (0.7)	0.5–1.2	2 (0.2)	0.1–0.9	14 (1.1)	0.6–1.8	7 (1.0)	0.5–2.1	7 (1.2)	0.6–2.4

Individuals with more than one NCD constituted 15% (95%CI: 14–17). Among adults, 59% (95%CI: 55–63) reported more than one NCD. Given the design, all households had at least one NCD patient; over half had more than one (53%, 95%CI: 48–59).

Twelve percent (251/2157, 95%CI: 10–3) of individuals had a physical disability requiring medical support.

### Awareness of Subsidized MoH Rates

Only 23% of interviewees (82/350, 95%CI: 19–28) were aware of the subsidized MoH rate for NCD patients and 7.1% (25/350, 95%CI: 4.9–10) knew that it is guaranteed until 2023.

Twenty-nine percent (100/250, 95%CI: 24–34) stated they will not seek MoH care after MSF closes, mainly due to the unaffordable price even with the subsidised rate (61%, 61/100, 95%CI: 51–70).

## Discussion

Our findings highlight increased socioeconomic and medical vulnerability among MSF Irbid Syrian refugee NCD patients and their households when compared to earlier studies.

### Demographic and Economic Characteristics

The proportion of adults (61%) is higher than the UNHCR estimate of 51% for Syrian refugees and asylum seekers registered in Jordan, the 46% found by the 2017 MSF survey of Syrian refugees residing in Irbid and the 56% estimate for the Jordanian population (19 years and older) [5, 7, 8]. Mean age in this study was higher than that found in the 2017 MSF survey (28.5 vs. 21.3 respectively). These differences in age, as well as other differences presented in this discussion, are likely to be explained by the fact that our study included only households with Syrian refugees residing in Irbid Governorate and attending MSF NCD services. Since NCD prevalence increases with age this would explain why this population is older. As a consequence of the increased age and NCD prevalence, increased healthcare needs and expenditures are expected.

The average household size of 6.2 is higher than the 5.9 average for Syrian refugees living in Jordan found by the Vulnerability Assessment Framework of Syrian Refugees in Jordan [9] and the 4.7 average household size in the Jordanian population [8], but lower than the 6.9 in the 2017 MSF survey [5].

Only 24% of adults were in paid work or self-employed. At household level, a high proportion (44%) had no working members. This is comparable to the findings of the Syrian refugees’ living conditions survey where just over half of households relied on work income [10]. For the 7.5% were in school/training, this likely reflects additional expense that should be queried further with respect to later employment/rewards.

The proportion of individuals registered with UNCHR in our study was similar to the 2017 MSF survey and the 2017/18 living conditions survey (96%, 95% and 97% respectively), while the proportion of MOI registration was higher (94% vs. 81% and 86% respectively) [5, 10]. This may indicate that registration status of MSF NCD patients has improved in recent years.

The mean household income was similar to the 2017 MSF survey and the 2017/18 living conditions survey (258 JOD, 239 JOD and 260 JOD respectively) [5, 10]; however, it was lower than the 376 JOD reported by the World Food Programme (WFP) 2018 assessment of Syrian refugees living in Jordan [11], and much lower than the 2017 national average reported by the Jordan Department of Statistics (889 JOD) [8]. Per capita income, given the average household size of 6.2 in our survey, is 42 JOD, which is lower than that of vulnerable Jordanians supported by the National Aid Fund (57 JOD) reported by WFP, and 38% lower than the existing 68 JOD poverty line [11].

Mean household expenditure was lower than that reported by the Jordan Department of Statistics for Jordanian national households (320 JOD vs 627 JOD) [8], and lower than in the earlier surveys (320 JOD vs. 359 JOD and 429 JOD respectively) [5, 10]. Although this indicates that mean debt was lower in our study, we found a higher proportion of households in debt compared with previous surveys, as 93% of households reported being in debt compared with the 79% in the 2017 MSF survey and 67% in the 2017/18 living conditions survey [5, 10].

The main source of household income in our study population was cash assistance (53%), a much higher proportion than the 31% in the 2017 MSF survey [12] and higher than the 39% reported for Syrian refugees in the 2018 WFP assessment [11]. In our survey, the second source of household income was work, reported by 43% of households; this was similar to the 45% reported by the 2017 MSF survey and lower than the 53% reported for Syrian refugees by the 2018 WFP assessment but higher than the 26% reported for vulnerable Jordanians [5, 11].

Almost 70% of households reported having to respond to an urgent health problem in the previous six months. Although we have not found other reports containing adequate indicators to compare this, the fact that a high proportion of households reported having to borrow money (60%), reduce standard household living spending (59%) or even sell household assets (18%) reveals their extreme financial vulnerability.

The proportion of people with physical disability (12%) was threefold higher than 3.9% found in the 2017 MSF survey [12]. It was even higher when compared to the 1.6% of people with a handicap/functional difficulty found by the 2017/18 living conditions survey, albeit using a different definition [10].

### Non-communicable Diseases

Comparison of self-reported NCD prevalence results with other (population-based) studies is limited by our redesign to include only MSF Irbid clinic patients and their households. However, the finding of 53% of households with more than one NCD patient was similar to a 2019 scoping review on the burden of non-communicable diseases among Syrian refugees [13].

The percentage of adults reporting at least one NCD under study (42%) was almost double the 22% in the 2017 MSF survey [5] and even higher than the 16% chronic health failure among Syrian refugees in Jordan reported in the 2017/18 living conditions survey [10]. Sixty-five percent of our patients had more than one NCD, which highlights the medical complexity and vulnerability of these people.

We found the most prevalent NCDs were hypertension and diabetes, aligning with findings of the 2017 MSF survey [5], the 2019 scoping review [13] and a recent study by Ratnayake, et al. [14]. We found higher prevalence of self-reported hypertension and diabetes (31% and 22% respectively) compared to the 2017 MSF survey (which found 14% and 9% respectively) [5] and the Ratnayake, et al. study (which found 17% and 10%) [14]. This is expected given our sampling strategy. The self-reported results are likely to be an underestimation of true biologically-based disease burden. The Ratnayake et al. study measured biologically-based prevalence to be approximately double the self-reported prevalence. Except for cardiovascular disease, NCD prevalence was higher among females, aligning with the 2017 MSF survey.

### Awareness of MoH Subsidized Healthcare

Awareness of the MoH subsidised rate for NCD care was low, with only 23% of interviewees being aware and only 7% being aware that it is guaranteed until 2023. This may indicate lack of access to appropriate and relevant information concerning health care rights or lack of information seeking in the MSF NCD cohort. Almost 30% of interviewees reported they would not seek MoH care after MSF closes; almost two thirds of these people stated the MoH subsidized price would still be unaffordable, highlighting their strained financial situation.

### Limitations

Our study included only households with Syrian refugees residing in Irbid Governorate and attending MSF NCD services, limiting generalizability to the full Irbid Syrian refugee population. Nonetheless, comparison of our results with those from previous surveys and available Syrian refugee and vulnerable Jordanian indicators aid understanding of this cohort’s characteristics and vulnerabilities.

All information collected was self-reported; we did not ask for proof. To decrease as much as possible the risk of lack of accuracy on the information provided by one person, the interviewers offered the interviewee the option of delegating or asking another household member to be present to assist specific responses if they were not sure about the answers. The interviewers were Jordanians who were trained on communication skills to make respondents feel comfortable. However, Syrian respondents may have perceived a difference in origin and dialect, which could have had an impact and potential bias in their responses.

Some interviewees may not have reported true household income and expenditure figures due to inaccurate calculations or preference for under- or overestimation. Debt accuracy and exchange mechanisms were not assessed in the data collection. Interviewees could have underreported individuals not registered with UNHCR or the MOI fearing legal repercussions. The research team was aware of these potential limitations and interviewers were trained to clearly state there were no incentives for survey participation and that all information remains anonymous.

## Conclusions

This study highlights increased vulnerability of MSF NCD patients in relation to the general Syrian refugee population residing in Jordan: this is an older population with high unemployment and reliance on cash assistance, high household debt and use of extreme coping mechanisms when facing a health emergency, and high multiple NCD comorbidity and physical disability. The continuous care that NCDs require creates an ongoing cost burden in households where expenditures already exceed income, risking further debt in households already living below the poverty line in Jordan.

Awareness of the MoH subsidised rate was low. This should be further explored as it may indicate lack of access to appropriate and relevant information concerning healthcare rights, lack of information seeking and/or other issues.

Almost one third of interviewees reported that if the free-of-charge MSF option is not available, they would not seek MoH care, most commonly stating it is unaffordable. These people are at increased risk of morbidity and mortality. It is vital that health actors providing care for Syrian refugees take action to reduce their risk, including making financial support mechanisms such as cash for health widely available and offering free healthcare.

## Data Availability

The datasets supporting the conclusions of this article are available from the corresponding author on reasonable request.
